# Ocular Pseudoexfoliation Syndrome and Vascular Disease: A Systematic Review and Meta-Analysis

**DOI:** 10.1371/journal.pone.0092767

**Published:** 2014-03-25

**Authors:** Wei Wang, Miao He, Minwen Zhou, Xiulan Zhang

**Affiliations:** Zhongshan Ophthalmic Center, State Key Laboratory of Ophthalmology, Sun Yat-Sen University, Guangzhou, People's Republic of China; Duke University, United States of America

## Abstract

**Objective:**

Many studies have assessed the association between ocular pseudoexfoliation syndrome (PEX) and vascular disease and produced controversial results. We performed a meta-analysis of epidemiologic studies to evaluate this relationship.

**Methods:**

Eligible studies that reported the incidence of vascular disease among PEX and control groups were identified via computer searches and reviewing the reference lists of the key articles. The summary odds ratio (OR) and 95% confidence interval (CI) were pooled using a random-effects model. Meta-regression to assess heterogeneity by several covariates and a subgroup analysis on study design and population were performed. Publication bias was tested by Begg's funnel plot and Egger's regression test.

**Results:**

Sixteen eligible studies involving 8,533 PEX patients and 135,720 control patients were included in the meta-analysis. All studies were performed primarily in whites with a mean age between 54.7 and 77.1 years. The overall combined ORs for patients with PEX compared with the reference group were 1.72 (95% CI: 1.31 to 2.26) for any vascular disease, 1.61 (95% CI: 1.22 to 2.14) for coronary heart disease, 1.59 (95% CI: 1.12 to 2.23) for cerebrovascular disease, and 2.48 (95% CI: 1.30 to 4.72) for aortic aneurysm. There was evidence of statistical heterogeneity; however, subgroup and sensitivity analyses showed this result to be robust. No evidence of publication bias was observed.

**Conclusions:**

The overall current literature suggests that PEX was associated with increased risk of vascular disease. Because of the limitations of the included studies and meta-analysis, the findings need to be confirmed in future research via well-designed cohort studies.

## Introduction

Pseudoexfoliation syndrome (PEX) is an age-related disorder characterized by the production and accumulation of an abnormal pseudoexfoliation fibrillar material in various ocular tissues [1]. This syndrome affects about 0.2–30% of people older than 60 years worldwide [2]. Ocular manifestations of PEX have been well defined, such as pseudoexfoliation glaucoma (PEG), cataract formation, zonular instability, etc. Pseudoexfoliation fibers also have been identified in many extra-ocular tissues, such as the heart, lung, gall bladder, kidney, and cerebral meninges, so the search for systemic implications of this syndrome has attracted a great deal of attention [3].

Vascular disease is the leading cause of death worldwide. In recent decades, a number of epidemiological and experimental studies have assessed the association of PEX with vascular disease risk [4], [5]. However, the results have been inconsistent. Some studies [6]–[15] have shown an association between PEX and increased systemic vascular risk, while others [16]–[21] have indicated the opposite. An improved understanding of this issue may have important public health and clinical implications given the possibility that slit-lamp examination of the eye for the diagnosis of PEX may identify individuals with an increased vascular disease risk [22]. With recently accumulating evidence, our goal, therefore, was to evaluate the association between PEX and the risk of vascular disease by conducting a systematic review and meta-analysis of all available epidemiological studies.

## Methods

This study was conducted using a predefined protocol and in accordance with the Preferred Reporting Items for Systematic Reviews and Meta-Analyses (PRISMA) Statement and the Meta-Analysis of Observational Studies in Epidemiology (MOOSE) guideline (Table S1) [23].

### 1. Search Strategy

The databases of PubMed, Embase, and Web of Knowledge were systematically searched for relevant articles published between 1966 and December 2013. Both medical search headings and open text fields were used to identify articles. No date or language restrictions were applied. The search terms for exposure were “pseudoexfoliation syndrome” and “exfoliative syndrome,” and the search terms for the outcomes were: “cardiovascular disease”, “coronary artery disease”, “myocardial infarction”, “heart attack”, “coronary heart disease”, “vascular disease”, “ischemic heart disease”, “ischaemic heart disease”, “stroke”, “transient ischemic attack”, “transient ischaemic attack”, “vascular accident”, “aneurysm”, and “cerebrovascular disease”. The search strategy was optimized for all consulted databases, taking into account the differences in the various controlled vocabularies as well as the differences of database-specific technical variations (e.g. the use of quotation marks). Once relevant articles were identified, their reference lists were searched for additional articles.

### 2. Inclusion and Exclusion Criteria

A study was considered relevant if it reported quantitative estimates of the unadjusted and (or) multivariable adjusted (i.e. age, sex, serum cholesterol, blood pressure, current smoking, diabetes, family history, etc.) odds ratio (OR) with a corresponding 95% confidence interval (CI) for the log relative risk for vascular events. As few studies were eligible and as authors employed heterogeneous endpoints related to vascular disease, we defined a composite of major clinical vascular disease s as the primary endpoint for our meta-analysis. Vascular diseases include coronary heart disease (CHD, such as myocardial infarction, angina pectoris, and other ischemic heart disease), cerebrovascular disease (CVD, such as cerebral hemorrhage and stroke), aortic aneurysm, and peripheral vascular disease. Unpublished papers, nonhuman studies, letters/case reports, studies enrolling <10 subjects or subjects age <18 years, editorials, reviews, studies lacking raw data, and studies lacking a suitable control group, and studies using inadequate case definition were excluded.

### 3. Data Extraction and Quality Assessment

Two reviewers independently extracted data using a standardized data-collection form, and any disagreements were discussed. The data collected included the first author, the year of publication, the study design, the population studied, the exposure and outcome evaluated, the number of cases and controls, the association measure, the point estimate and 95% CI, and any adjustment/stratification/matching variables. In studies with overlapping patients or controls, only the latest or the most complete were included.

The qualities of included case-control studies were assessed independently by the same two investigators using the Newcastle-Ottawa Scale (NOS) [24]. The NOS uses a “star” rating system to judge quality based on three aspects of the study: selection, comparability, and exposure. The scores ranged from 0 star (worst) to 9 stars (best). Studies with a score ≥7 were considered to be of high quality. Disagreements were settled as described above.

### 4. Data synthesis and analyses

The pooled ORs with 95% CIs were calculated with the method of DerSimonian and Laird using the assumptions of a random-effects model to provide a conservative estimate of the effect owing to potential population differences among the studies. Where possible, data for maximally adjusted risk estimates were extracted. In studies where no appropriate association measure was provided, if possible, the raw aggregated data (ignoring matching designs, where necessary) were used to estimate unadjusted associations.

Heterogeneity was assessed using the Q statistic and the I^2^ statistic. An I^2^ value of more than 50% was defined to represent obvious heterogeneity. For the Q statistic, a P value of <0.1 was considered statistically significant heterogeneity. To examine the magnitude of the combined OR in each stratum and its respective test of heterogeneity, subgroup analyses according to study design, geographic area, study quality, number of cases, and levels of adjustment were conducted. Meta-regression analysis was used to investigate the influence of these variables on study heterogeneity across strata. We further conducted a sensitivity analysis to explore possible explanations for heterogeneity and to examine the influence of various exclusion criteria on the overall risk estimate. We also investigated the influence of a single study on the overall risk estimate by omitting one study in each turn.

Potential publication bias was assessed by visual inspection of Begg funnel plots in which the log ORs were plotted against their SEs. We also performed the Begg rank correlation test and Egger linear regression test at the p<0.10 level of significance. All analyses were performed using Stata version 12.0 (StataCorp LP, College Station, Texas). A P-value <0.05 was considered statistically significant, except where otherwise specified.

## Results

### 1. Literature search

**Figure 1:**
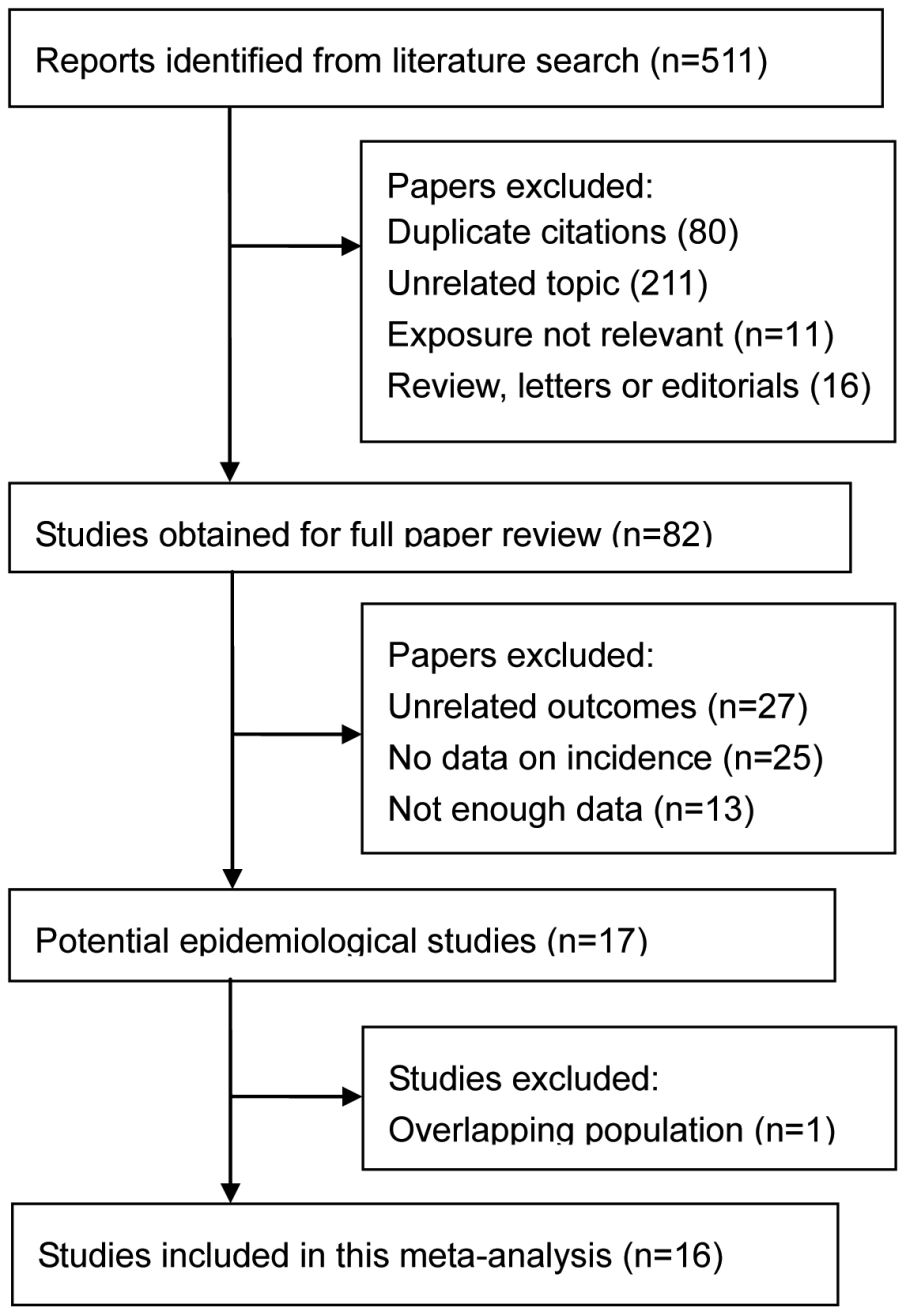
Diagram of study selection and inclusion.

Upon the literature search and manual review for pertinent references, 511 citations were retrieved and evaluated for inclusion at the title and abstract review stage (Fig. 1). From these, 82 full-text articles were evaluated for inclusion. Sixty-five articles were excluded because they did not assess the association between PEX and vascular disease, provided no data on vascular disease incidence, had insufficient data, or were duplicate publications. Finally, sixteen studies [6]–[21] were included in this meta-analysis.

### 2. Study characteristics

The main characteristics of the studies included in this analysis are provided in Table 1. Agreement between the 2 reviewers was 95% for data extraction and 93% for quality assessment of trials. Among the 16 identified articles, seven were population-based and nine were hospital-based studies. Five studies were done in Turkey, two each in India, Greece, and Norway, and one each in Lithuania, the U.S.A., Spain, Serbia, and Australia, involving a total of 8,533 PEX patients and 135,720 control patients. All studies were performed primarily in whites with a mean age between 54.7 and 77.1 years. The research outcome varied and mainly included CHD, angina, myocardial infarction, CVD, and aortic aneurysm, collected from self-report questionnaires, medical records, or national registries. Sixteen studies investigated associations of PEX and total vascular disease, 14 studies investigated PEX and CHD, 4 studies investigated PEX and CVD, and 3 studies investigated PEX and aortic aneurysm. One study adjusted for age only, and 8 studies further controlled a group of conventional risk factors (sociodemographics, comorbidites, and lifestyle factors) for vascular disease, whereas others adjusted no factors. The NOS results showed that the average score was 7.5 (range 7 to 9), indicating that the methodological quality was generally good.

**Table 1:** Characteristics of studies included in the meta-analysis.

First author (year)	Country	Design	No. of eyes	Age (year)	Sex (M/F)	Covariates adjusted
Kocabeyoglu (2013)[6]	Turkey	CC, HB	40/40	69.6/67.1	27/13; 24/16	Age, gender, place of residence
Gonen (2013)[7]	Turkey	CS, HB	49/42	71.1/69.5	27/22; 20/22	None
French (2012)[8]	U.S.A.	CS, PB	6,046/125,924	77.1/71.8	5,853/193; 125,924/0	Age, race, gender, hypertension, hyperlipidemia, diabetes, alcohol use, and tobacco exposure
Djordjevic-Jocic (2012)[9]	Serbia	CS, HB	60/60	69.6/67.9	22/38; 21/39	Age, gender
Praveen (2011)[10]	India	CC, HB	40/120	69.0/68.83	82/78	Age, sex
Andrikopoulos (2009)[11]	Greece	CS, HB	596/1544	71.4	1,088/1,052	Age, serum cholesterol, blood pressure, current smoking, diabetes, family history of premature CAD, and obesity
Sekeroglu (2008)[12]	Turkey	CC, PB	242/1238	74.3/66.5	116/126; 584/654	Age
Citirik (2007)[13]	Turkey	CC, HB	40/60	60.57/56.92	18/22; 23/37	Age, sex, diabetes mellitus
Ritland (2004)[14]	Norway	CS, PB	718/429	NA	438/280; 234/195	None
Mitchell (1997)[15]	Australia	CS, PB	81/3465	NA	NA	None
Speckauskas (2012)[16]	Lithuania	CS, PB	152/193	61.8/67.0	67/85; 352/561	None
Viso (2010)[17]	Spain	CS, PB	55/564	73/59	26/29; 203/361	None
Emiroglu (2010)[18]	Turkey	CC, HB	24/466	65.9/54.7	NA	None
Tarkkanen (2008)[19]	Norway	CS, PB	155/344	73/69	56/109; 117/227	Age, sex
Brajkovi (2007)[20]	India	CC, HB	161/485	68.42	297/349	None
Konstas (1998)[21]	Greece	CC, HB	74/26	68.3/62.4	48/26; 16/10	Age, sex, glaucoma, systolic pressure, diabetes, body-mass index, serum cholesterol, and current smoking

CC = case-control study; CS =  cross-sectional study; HB = hospital-based; PB = population-based; NA = not available.

### 3. Association between PEX and vascular disease

**Figure 2:**
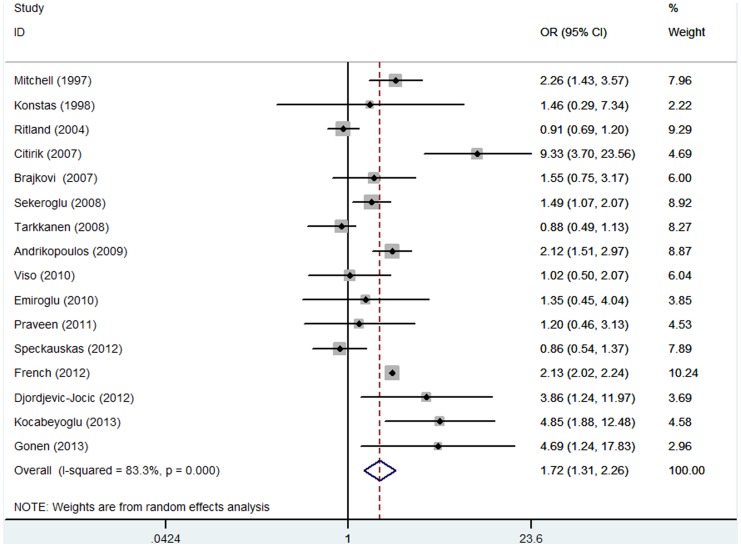
Random-effects meta-analysis of studies that examined PEX and risk of vascular disease.

Figure 2 shows the forest plot of the association between PEX and vascular disease. Overall, patients with PEX, compared with the control group, experienced a significantly increased risk for developing vascular disease [OR: 1.72 (95% CI: 1.31 to 2.26); p<0.001]. Substantial heterogeneity was observed (P<0.001, I^2^ = 83.3%). When we evaluated the relation of PEX with risk of CHD and angina, the associations were all statistically significant, with pooled OR at a 95% CI of 1.61 (1.22 to 2.14) and 1.71 (1.34 to 2.18), respectively. As for CVD, for four studies, the summary OR at a 95% CI was 1.59 (1.12 to 2.23), with no evidence of heterogeneity (P = 0.314, I^2^ = 15.5%). With respect to aortic aneurysm, the pooled OR was 2.48 (95% CI: 1.30 to 4.72), with no significant heterogeneity (P = 0.154, I^2^ = 46.5%).

### 4. Subgroup, sensitivity analyses, and meta-regression

To explore further the heterogeneity among studies of PEX and vascular disease, we performed subgroup and sensitivity analyses. When a subgroup analysis was stratified by research design, source of control, sample method, number of cases, geographic region, age, gender, publication year, and adjustment variables, a positive relation between PEX and vascular disease risk was observed in all subgroups except in the POAG subgroup as control patients (Table 2). There was evidence of heterogeneity in all subgroups except in POAG subgroups as control patients and studies conducted outside Europe. Additional analyses assessed robustness to examine the influence of various exclusion criteria on the overall risk estimate. Furthermore, the sensitivity analyses that omitted one study at a time and calculated the combined OR for the remaining studies yielded consistent results. The combined ORs were all statistically significant and similar with one another, with a narrow range from 1.75 (95% CI: 1.42 to 2.16) to 2.00 (95% CI: 1.62 to 2.48). In meta-regression analysis, we explored the influence of key characteristics of the studies (subgroup factors) on heterogeneity (Table 2). Only the number of cases was found to contribute to substantial heterogeneity (P = 0.034).

**Table 2:** Combined OR of vascular disease related to PEX by study design and population characteristics.

		N	OR (95% CI)	*P* for heterogeneity	*I ^2^* (%)	P	P for meta-regression
All studies		16	1.72 (1.31, 2.26)	<0.001	83.30	<0.001	-
Disease type							-
	CHD	14	1.61 (1.22, 2.14)	<0.001	85.00	0.001	
	Angina	3	1.71 (1.34, 2.18)	0.328	10.30	<0.001	
	CVD	4	1.59 (1.12, 2.23)	0.314	15.50	0.009	
	Aneurysm	3	2.48 (1.30, 4.72)	0.154	46.50	0.006	
Study design							0.290
	Cross-sectional	9	1.59 (1.19, 2.13)	<0.001	86.30	0.002	
	Case-control	7	2.20 (1.42, 3.39)	0.037	55.20	<0.001	
Source of control							0.119
	POAG as control	3	1.17 (0.94, 1.46)	0.881	0.00	0.163	
	Non-POAG as control	13	1.95 (1.56, 2.44)	<0.001	68.5	<0.001	
Sample method							0.258
	Hospital-based	11	2.21 (1.46, 3.35)	0.042	54.10	<0.001	
	Population-based	7	1.58 (1.16, 2.14)	<0.001	86.50	<0.001	
Number of cases							0.034
	<100	8	2.67 (1.72, 4.16)	0.076	47.60	<0.001	
	≥100	8	1.47 (1.12, 1.92)	<0.001	86.10	0.005	
Geographic region							0.774
	Europe	11	1.74 (1.26, 2.40)	<0.001	75.60	0.001	
	Others	5	2.12 (2.01, 2.23)	0.697	0.00	<0.001	
Age							0.596
	<Median	8	1.98 (1.09, 3.62)	0.001	73.50	0.026	
	≥Median	8	1.90 (1.54, 2.35)	0.023	64.70	<0.001	
Male of cases							0.932
	<50%	8	1.89 (1.13, 3.17)	<0.001	82.90	0.016	
	≥50%	8	1.77 (1.33, 2.37)	<0.001	78.10	<0.001	
Publication year							0.797
	Before 2009	7	1.71 (1.19, 2.46)	0.001	72.20	0.004	
	After 2009	9	1.86 (1.41, 2.46)	0.004	68.30	<0.001	
Adjustment for confounding factors							0.290
	Any	9	1.99 (1.56, 2.53)	0.007	64.00	<0.001	
	None	7	1.50 (1.04, 2.17)	0.008	67.90	0.028	
	Age, sex	4	1.96 (1.07, 3.61	0.105	51.20	0.030	
	Age, sex, DM	3	3.21 (1.10, 9.38)	0.007	80.00	0.033	
	Age, DM and other vascular risk factors	3	2.11 (2.01, 2.22)	0.449	0.00	<0.001	

PEX = Pseudoexfoliation syndrome; CHD =  Coronary heart disease; CVD =  Cerebrovascular disease; DM =  Diabetes mellitus.

### 5. Publication bias

Visual inspection of the Begg funnel plot did not identify substantial asymmetry. The Begg rank correlation test and the Egger linear regression test also indicated little evidence of publication bias among studies of PEX and vascular disease risk (Begg, P = 0.274; Egger, P = 0.280).

## Discussion

Pseudoexfoliation syndrome is easily diagnosed by slit-lamp examination on anterior segment changes—whitish deposits on the pupillary margin or anterior side of the lens, along with heavy chamber angle pigmentation. For more than a decade, the scientific debate on the relationship between PEX and systemic vascular disease risk has remained unsettled [25]. To our knowledge, this is the first meta-analysis investigating the association between PEX and vascular disease. Our results showed that PEX increased the odds of vascular disease by 72%, so the detection of PEX during routine ophthalmologic examination could be an important indicator of risk for systematic vascular disease. This finding is consistent with those reported for other health outcomes, such as all-cause mortality.

The results from our subgroup and sensitivity analyses were quite similar and robust, and the associations were neither significantly modified by research design, source of control, sample method, number of cases, geographic region, age, gender, publication year, or adjustment variables nor substantially driven by any single study. A significant positive association was observed in all subgroups except in the subgroup using POAG as control. Although vascular risk may differ between PEX and POAG patients, the non-significant association in the subgroup using POAG as controls was likely due to the small number of studies (n = 3) and, hence, insufficient statistical power. As for individual studies, several studies differed from others in various aspects. For example, in the study by Mitchell et al [15], the oldest one, published in 1997, no adjustment was conducted; the study by Kocabeyoglu et al [6], the most recent one, enrolled only 40 cases and 40 control patients; and the study by French et al [8] was the largest study, accounting for 10.24% of the total weight in the current meta-analysis. Nevertheless, the combined risk estimate was not significantly driven by any single study in the sensitivity analysis.

The underlying mechanisms linking PEX to vascular disease are not fully understood, but several possible biologic mechanisms have been suggested. The accumulation of pseudoexfoliation material in various tissues seen with ageing is one of the proposed causal mechanisms [26]. The pericellularly accumulating pseudoexfoliation material may disrupt the normal basement membrane of the cells and cause degenerative fibrillopathy [5]. Furthermore, these deposits may lead to impaired endothelial function. Endothelial dysfunction is an independent predictor of future vascular events. Some studies have experimentally showed the increased concentration of endothelin-1, a potent vasoconstrictor, in the plasma and aqueous humour in PEX patients [27]. This process may, in due course, result in weakened elasticity and contractility of vascular wall muscles and increased vascular resistance. There is evidence that PEX patients had elevated plasma homocysteine compared to healthy controls. Some evidence has also suggested that an excess level of homocysteine may induce neural cell death and degradation of the elastic structures in the arterial wall [28]. In addition, some studies have shown significantly high levels of hydrogen peroxide and xanthine oxidase, in contrast with a lower level of catalase and paraoxonase activities, suggesting increased oxidative stress and decreased total antioxidant capacity in PEX patients [29]. Other possible mechanisms include an overexpression of the basic fibroblast growth factor, an imbalance in the matrix metalloproteinases (MMPs)/tissue inhibitors of MMPs, and increased serum antiphospholipid antibody levels [30], [31]. Therefore, further experimental and clinical studies are needed to elucidate potential pathways of effects of PEX on vascular disease.

Our analysis provides more precise estimates for vascular disease compared with prior observations by using a large pool of studies and using adjusted estimates. The current meta-analysis is also consistent with clinical reports showing an association between ocular PEX and hemodynamic parameters. Recently, Ulus et al [25] demonstrated that myocardial peak systolic velocities were lower and carotid intima-media thickness was increased in patients with PEX compared with age-matched and sex-matched controls. Another study showed through transcranial Doppler imaging that blood flow velocity was reduced and the resistance of the middle cerebral arteries was elevated in PEX patients in comparison to age- and sex-matched controls [32]. Other studies have demonstrated impairment of cardiovagal regulation and impairment of conduit artery function in PEX patients [26]. The decrease in blood flow velocities might predispose the patient to vascular diseases and might be a risk marker for systemic vascular disease.

Substantial heterogeneity was observed among studies of PEX and vascular disease risk, which was not surprising given the differences in the characteristics of populations, ascertainment of vascular disease, and adjustment for confounding factors. Nonetheless, we failed to find the major source of study heterogeneity in a sensitivity analysis. In a meta-regression analysis, the number of cases was statistically significant. Interestingly, there was still substantial heterogeneity among studies with the number of cases exceeding 100; an explanation might be that authors used different statistical approaches and adjusted for different potential confounding variables. Of note, seven out of sixteen studies calculated only crude ORs rather than a multivariable analysis controlling for relevant confounders. All of these factors could be relevant when considering the difference in findings. Further evaluation of effect modification is needed in larger studies or individual participant pooled analyses with more power to detect effect modification than our analyses based on study-level characteristics. In addition, since only a few studies were eligible, we pooled results from studies involving different designs and dissimilar definitions of vascular disease. However, nearly all of the papers that were finally included in this meta-analysis were ones that generally had a positive correlation between vascular disease and pseudoexfoliation syndrome.

A major strength of our study is that a comprehensive and transparent literature search identified all relevant reports, and the methodological quality of included studies was assessed according to standard and detailed criteria. The inclusion of more than 100,000 control patients and 8,000 PEX cases greatly enhanced the statistical power to provide more precise and reliable risk estimates. Moreover, the association of PEX with vascular disease persists and remains statistically significant in subgroup analysis according to key characteristics and sensitivity analyses based on various exclusion criteria.

Limitations in this study should also be emphasized. First, the majority involved retrospective data analyses, which increase the risk of bias associated with the recording of baseline data, the need for imputation, and potential selection bias. The appropriateness of the reference group is of paramount importance, and the ideal comparison group should be similar to the PEX in all ways except in their ocular signs. The ORs of vascular disease and PEX were least when compared to a population of patients with POAG, because POAG itself a risks factor for vascular disease. Individuals with PEX are more likely to seek medical attention and be screened for vascular disease; thus, the recorded increased incidence may be an artifact of more complete detection rather than PEX itself driving the effect. Second, the included studies are not equally designed, and obvious heterogeneity was detected across our study. Although we did not identify the sources of heterogeneity, our subgroup and sensitivity analyses suggest that the results were consistent and robust. Third, residual confounding is of concern. Uncontrolled or unmeasured risk factors potentially produce biases. Although subgroup analyses by adjustment for potential confounders did not materially alter the combined risk estimate, we still cannot rule out the possibility that residual confounding could affect the results because these factors do not explain all of the risk for vascular events. Fourth, because current data in relation to PEX and outcomes for vascular disease are sparse, we were unable to investigate each subtype of vascular disease. Fifth, we cannot eliminate publication bias, though neither Begg's test nor Egger's test suggests publication bias. Finally, most studies were conducted by European and North American populations, with predominately Caucasian participants. It is possible that the strength or existence of the association varies by ethnicity. Therefore, more studies especially well-designed cohort studies are warranty to confirm the findings in this analysis.

On the basis of our findings, several questions arise. The first is whether PEX has a causal effect on vascular disease or is only a surrogate marker for other biological risk factors. To answer this question, several issues should be considered, including the use of a standardized vascular disease definition, the interval between the incidence of the two diseases, and adequate control for confounding factors. Second, by what exact mechanisms does PEX increase the risk of vascular disease? Expanding the number of newly recognized, potentially independent risk factors for vascular disease, such as inflammation (as measured by the C-reactive protein), vitamin D level, and hours of regular sleep may be advantageous [33]. Further studies, including well-designed clinical trials and cohort studies, are warranted to address these questions for a better understanding of the association and to provide convincing evidence for clinical practice in systematic vascular disease prevention.

### Conclusions

The findings of this meta-analysis suggest that PEX is associated with an increased risk of vascular disease. Because of the limitations of the included studies and meta-analysis, subsequent well-designed prospective cohort studies that adequately control for confounding factors and detection bias are urgently needed to confirm our results. The underlying mechanisms that link PEX to vascular disease also deserve further investigations.

## Supporting Information

Table S1 **PRISM Checklist.**
(DOC)
