# Supplementary material for: Ocular Pseudoexfoliation Syndrome and Vascular Disease: A Systematic Review and Meta-Analysis
Source: PLoS One. 2014 Mar 25;9(3):e92767. doi: 10.1371/journal.pone.0092767 (PMC3965457; doi:10.1371/journal.pone.0092767)
Supplement: Table S1 — PRISM Checklist. (DOC) [file pone.0092767.s001.doc]

**Table S1. MOOSE Checklist**

| **Criteria** | | **Page** |
| --- | --- | --- |
| **Reporting of background should include** | |  |
| √ | Problem definition | 3 |
| √ | Hypothesis statement | 3 |
| √ | Description of study outcomes | 3 |
| √ | Type of exposure or intervention used | 3 |
| √ | Type of study designs used | 3 |
| √ | Study population | 3 |
| **Reporting of search strategy should include** | |  |
| √ | Qualifications of searchers | 3 |
| √ | Search strategy, including time period included in the synthesis and keywords | 4 |
| √ | Databases and registries searched | 4 |
| √ | Search software used, name and version, including special features | 4 |
|  | Use of hand searching | 4 |
| √ | List of citations located and those excluded, including justifications | 4 |
|  | Method of addressing articles published in languages other than English | 4 |
| √ | Method of handling abstracts and unpublished studies | 4 |
| √ | Description of any contact with authors | 4 |
| **Reporting of methods should include** | |  |
| √ | Description of relevance or appropriateness of studies assembled for assessing the hypothesis to be tested | 4 |
| √ | Rationale for the selection and coding of data | 4 |
| √ | Assessment of confounding | 5 |
| √ | Assessment of study quality, including blinding of quality assessors; stratification or regression on possible predictors of study results | 5 |
| √ | Assessment of heterogeneity | 5 |
| √ | Description of statistical methods in sufficient detail to be replicated | 6 |
| √ | Provision of appropriate tables and graphics | 6 |
| **Reporting of results should include** | |  |
|  | Graph summarizing individual study estimates and overall estimate | 6 |
| √ | Table giving descriptive information for each study included | 7 |
| √ | Results of sensitivity testing | 8 |
| √ | Indication of statistical uncertainty of findings | 8 |
| **Reporting of discussion should include** | |  |
| √ | Quantitative assessment of bias | 9-14 |
| √ | Justification for exclusion | 9-14 |
| √ | Assessment of quality of included studies | 9-14 |
| **Reporting of conclusions should include** | |  |
| √ | Consideration of alternative explanations for observed results | 14 |
| √ | Generalization of the conclusions | 14 |
| √ | Guidelines for future research | 14 |
| √ | Disclosure of funding source | 1 |
